# RADTHYR: an open-label, single-arm, prospective multicenter phase II trial of Radium-223 for the treatment of bone metastases from radioactive iodine refractory differentiated thyroid cancer

**DOI:** 10.1007/s00259-021-05229-y

**Published:** 2021-02-23

**Authors:** Désirée Deandreis, Aline Maillard, Slimane Zerdoud, Claire Bournaud, Lavinia Vija, Christophe Sajous, Marie Terroir, Laurence Leenhardt, Martin Schlumberger, Isabelle Borget, Sophie Leboulleux

**Affiliations:** 1 Nuclear Medicine and Endocrine Oncology, Gustave Roussy and Paris Saclay University, 114 Rue Edouard Vaillant, Villejuif, France; 2 Present Address: Department of Medical Sciences, University of Turin, Corso Dogliotti, 14, 10126 Torino, Italy; 3 Department of Biostatistics and Epidemiology, Gustave Roussy, University Paris-Saclay, 114 Rue Edouard Vaillant, Villejuif, France; 4 Oncostat U1018, Inserm, University Paris-Saclay, labeled Ligue Contre le Cancer, 114 Rue Edouard Vaillant, Villejuif, France; 5 Nuclear Medicine, Institut Universitaire du Cancer Toulouse Oncopole, 1 Avenue Hubert Curien, Toulouse, France; 6 Nuclear Medicine, Hospices Civils de Lyon, Groupement Hospitalier Est, 59 boulevard Pinel, Bron, France; 7 Department of Medical Oncology, Hospices Civils de Lyon, Groupement Hospitalier Est, 59 boulevard Pinel, Bron, France; 8 Thyroid and Endocrine Tumors Unit, Pitié-Salpêtrière Hospital Sorbonne University, 47-83 Boulevard de l’Hôpital, Paris, France

**Keywords:** Radium-223, Refractory thyroid cancer, Bone metastases, Alpha emitters, Leukemia

## Abstract

**Purpose:**

This is the first prospective trial evaluating the efficacy of alpha emitter Radium-223 in patients with bone metastases from radioactive iodine (RAI) refractory (RAIR) differentiated thyroid cancer.

**Methods:**

RADTHYR is a multicenter, single-arm prospective Simon two-stage phase II trial (NCT02390934). The primary objective was to establish the efficacy of three administrations of 55 kBq/kg of Radium-223 by ^18^F-FDG PET/CT according to PERCIST criteria. Secondary objectives were to establish the efficacy of six administrations of Radium-223 by ^18^F-FDG PET/CT, ^99m^Tc-HMDP bone scan and ^18^FNa PET/CT, clinical benefits, changes in serum bone markers, thyroglobulin levels, and safety.

**Results:**

Ten patients were enrolled between July 2015 and December 2017 (4 M; median age 74 years). Prior to Radium-223 administration, patients received a median RAI cumulative activity of 15 GBq (7.4–35.6), external radiation therapy (*n* = 9), bone surgery (*n* = 8), cimentoplasty (*n* = 5), and cryoablation (*n* = 2). ^18^F-FDG PET/CT showed stable disease (SD) in 4/10 and progressive disease (PD) in 6/10 cases after three administrations and SD in 4/10, PD in 5/10 cases, and 1/10 non-evaluable (NE) case after six administrations. After six injections, ^99m^Tc-HMDP bone scan showed SD in 9 cases and was NE in 1 case; ^18^FNa PET/CT showed SD in 8 cases, partial response (PR) in 1 case, and was NE in 1 case. No significant clinical benefits were reported during the study. A skeletal event occurred in 6 patients (median time without skeletal event of 12.1 months). Seventy-seven adverse events were reported during treatment (7 of grade 3–4). Three patients developed an acute myeloid, a promyelocytic, and a chronic myeloid leukemia after the last Radium-223 administration considered as drug-related.

**Conclusion:**

The trial was stopped after interim analysis for lack of response of bone metastases from RAIR thyroid cancer to Radium-223. Severe hematological toxicity was observed in patients heavily pretreated with RAI and external radiation.

**Trial registration number:**

NCT02390934. Registration date 18.03.2015.

**Supplementary Information:**

The online version contains supplementary material available at 10.1007/s00259-021-05229-y.

## Introduction

Distant metastases occur in 4 to 10% of patients with differentiated thyroid cancer (DTC) [1]. When present, the 5-year survival ranges from 78% in patients with single-organ metastasis to 15% in patients with multi-organ metastases [1–3]. Radioactive iodine (RAI) remains the milestone for the treatment of patients with distant metastases and a prompt identification is necessary. Bone lesions are present in 20–40 % of patients with distant metastases; they can be synchronous or metachronous to primary thyroid tumor diagnosis and are the most challenging metastases to treat [4]. They can be lytic, sclerotic, or mixed with a combined lytic and sclerotic aspect on imaging. In cases of small size lesions and in the presence of RAI uptake, complete response can be achieved [5]. However, bone lesions are often large with soft tissue infiltration, poor response to RAI, or progressive loss of RAI uptake with subsequent worst prognosis especially when present since the beginning of the clinical history [4, 6]. Local treatments such as surgery, external beam radiation, thermoablation, and cement injection may prevent skeletal-related events as well as bone resorption inhibitors such as denosumab or bisphosphonates [7–9]. However, the rapid occurrence of bone events remains frequent, occurring in one third of patients with bone metastases, highlighting the need for specific bone treatments [10].

Radium-223 is an alpha emitter radionuclide with a biological effect characterized by DNA double-strand breaks with cell apoptosis and a high cytotoxic effect. It shares similar chemical properties with calcium, and its uptake in bone matrix is correlated with bone remodeling. Radium-223 (Xofigo®) was approved by the Food and Drug Administration (FDA) and by the European Medical Agency (EMA) for the treatment of symptomatic bone metastases from castration-resistant prostate cancer, based on improvement in overall survival in a phase III trial [11]. Patients with prostate cancer mostly have sclerotic bone lesions, a favorable setting for the efficacy of Radium-223, since it preferably accumulates in these lesions with a subsequent good control of pain and longer PFS especially in patients with low tumor burden [12]. However, preliminary data on breast cancer showed its potential effectiveness in a setting of mixed and lytic lesions [13–16].

The aim of this phase II trial was to evaluate for the first time the efficacy and the tolerance of Radium-223 treatment in patients with bone metastases from RAI refractory (RAIR) DTC and not candidate for target therapy or local treatments.

## Materials and methods

### Study design

The RADTHYR study was a prospective, multicenter, open-label phase II trial (ClinicalTrials.gov number NCT02390934) of Radium-223 in patients with bone metastases from RAIR DTC approved by the local institutional board of Gustave Roussy, Institut Universitaire du Cancer Toulouse Oncopole and Groupement Hospitalier Est of Bron. The primary objective was to evaluate the efficacy of three monthly Radium-223 administrations by ^18^F-Fluorodeoxyglucose positron emission tomography/computed tomography (^18^F-FDG PET/CT) according to PET Response Criteria In Solid Tumor (PERCIST) [17].

The secondary objectives were to (1) evaluate the efficacy of six monthly administrations of Radium-223 by ^18^F^-^FDG PET/CT according to PERCIST criteria; (2) evaluate monthly the clinical benefits during treatment; (3) evaluate the time to occurrence of skeletal events; (4) evaluate the response on ^99m^Tc-hydroxymethylene diphosphonate (HMDP) bone scan and on sodium-fluoride (^18^FNa) PET/CT after three and six monthly administrations of Radium-223; (5) describe monthly changes in serum alkaline phosphatase (ALP), bone-ALP (b-ALP), and thyroglobulin (Tg) during Radium-223 administration; and 6) assess the safety of Radium-223 according to NCI CTCAE version 4.

### Patient selection

The main inclusion criteria were as follows: (1) histologically confirmed DTC (papillary, follicular, Hurthle cell, or poorly differentiated); (2) metastatic RAIR refractory disease (absence of RAI uptake in metastatic lesions or in case of RAI uptake present in some but not in other tumor foci or progression of the disease within 14 months after RAI administration treatment or persistent disease after the administration of a cumulative activity of 22 GBq; (3) age ≥ 18 years; (4) Eastern Cooperative Oncology Group (ECOG) performance status 0–2; (5) presence of at least one bone metastasis visible on CT scan and not needing imminent local treatment; (6) stable disease within 6 months before inclusion and low likelihood of an indication for systemic treatment within the next 6 months; (7) absence of visceral metastases or visceral metastases stable within 6 months before inclusion according to morphological imaging; (8) presence of at least one bone metastasis with uptake on ^18^F-FDG PET/CT; (9) presence of at least one bone metastasis with increased uptake on ^99m^Tc- HMDP bone scintigraphy or on ^18^FNa PET/CT; and (10) adequate hematological parameters. Patients receiving bisphosphonates or anti-receptor activator of nuclear factor κ (RANK) ligand were allowed but patients should have received at least two administrations prior to Radium-223 administration, and these treatments were continued during Radium-223 treatment. The complete lists of inclusion and exclusion criteria are presented in Supplementary Table 1.

### Protocol

Radium-223 (55kBq/kg, IV) was administered according to the international guidelines every 4 weeks for 3 months [18]. In the absence of severe adverse events, skeletal-related events, or appearance of new lesions at ^18^F^-^FDG PET/CT, further three monthly Radium-223 injections were administered. If only an increase of uptake of ^18^F-FDG occurred in the existing lesions after three administrations of Radium-223, the treatment was continued.

A functional and morphological evaluation (including an ^18^F-FDG PET/CT, ^99m^Tc-HMDP bone scan, ^18^FNa PET/CT) was performed within 4 to 8 weeks prior to Radium-223 treatment initiation, and then 1 month after the third and the sixth administration. Clinical and biological (hematology, biochemistry, and thyroglobulin) evaluations were performed each month before Radium-223 administration to assess the clinical benefits and toxicity. After the last Radium-223 treatment, patients were followed every 3 months until 12 months. Patients performed all PET/CT and bone scintigraphies in the same center. Radium-223 continuation was decided locally while all PET images (both ^18^F-FDG and ^18^FNa) and bone scans were centrally reviewed by the same expert physician in nuclear medicine (D.D).

### Imaging protocols

^18^F-FDG PET/CT was performed after a 6-h fast, in case of plasma glucose level < 10mmol/L prior to ^18^F-FDG injection, with administration of a maximal ^18^F-FDG activity of 3.5 MBq/kg. PET images were performed 60 ± 10 min after tracer injection and recorded from the apex of the skull to the mid-thighs in 3D mode, 7–10 bed, 2 min/bed position. ^18^FNa PET/CT images were performed 60 ± 10 min after injection of a maximal activity of 3.5 MBq/kg and whole-body images were recorded in 3D mode, 7–10 bed, 2 min/bed position. Prior to PET images a whole-body low-dose CT acquisition for attenuation correction and anatomical correlation was performed. Images were reconstructed using an iterative algorithm (FORE and AWOSEM) and a time of flight (TOF) technique. ^99m^Tc HMDP Bone scan was performed with anterior/posterior whole-body planar images recorded on a double-headed gamma camera 3 h after the administration of 10 MBq/kg and additional single photon emission tomography (SPECT/CT) was carried out in indeterminate abnormalities. For each patient, both ^18^F-FDG PET and ^18^FNa PET and bone scintigraphy were performed on the same scanner and with the same time/activity protocol (Discovery 690 PET/CT and Discovery 670 SPECT/CT, GE Healthcare for Gustave Roussy, GE Discovery IQ and GE Discovery NM CT 670 GE Healthcare for Toulouse; Siemens Biograph mCT 64 and Symbia T2 SPECT/CT for Bron). The PET/CT scanners employed in the study had an active EARL accreditation in all centers.

### Evaluation criteria

A detailed summary of the evaluation criteria is available in Supplementary Table 2. Because RECIST 1.1 does not apply to bone evaluation, response evaluation was based on metabolic response assessed 1 month after three monthly (primary objective) and six monthly (secondary objective) injections of Radium-223 on ^18^F-FDG PET/CT according to PERCIST [17]. Lean body mass corrected total standardized uptake value (SUL) peak in up to five lesions with the highest ^18^F-FDG uptake was considered the main criteria for disease response. The total lesion glycolysis (TLG) defined as SUVmean × functional volume (FV) was considered for progressive disease (PD) definition as proposed by PERCIST. Pure bone lesion without extension to soft tissue, without metallic part, not previously treated with radiotherapy or focal treatment were considered target lesions. The total SUVmax was considered the main criteria for functional changes assessment in up to five lesions with the highest ^18^FNa PET/TC uptake. All lesions were manually segmented by drawing a spherical volume of interest (VOI) and the semiquantitative parameters were extracted by an automated dedicated multivendor software for imaging analysis. A 40% threshold was settled for SUVmax. For ^99m^Tc HMDP bone scan evaluation, a visual analysis was performed.

Clinical benefits were evaluated monthly, and data were compared to baseline examinations. These included pain response, based on the numerical visual analogue pain scale rating from 0 to 10 completed by the patient; furthermore, the improvement of ECOG performance status was considered. Serum thyroglobulin (Tg) in the absence of Tg antibodies (Ab), alkaline phosphatase (ALP), and bone-ALP changes was determined monthly and compared to baseline values. The skeletal-related event definition included local progression with indication for focal treatment and/or pathological fracture and/or spinal cord compression. Adverse events and their grade were assessed according to the National Cancer Institute Common Terminology Criteria for AEs, version 4.0. Twelve months of safety monitoring after the last administration were planned for all patients who received at least one Radium-223 injection.

### Statistical analysis

The study was conducted according to a Simon minimax two-stage design to allow early termination of the trial for lack of efficacy [19]. Sample size was calculated on the hypothesis that a probability of 15% was the minimal response rate and a probability of 40% was the expected response rate, with a power of 80% and a type I error rate of 5%. Nine patients had to be enrolled in the first stage, and the study continued to 19 patients only if at least two of the nine patients showed a metabolic response. At the end of the study, if six metabolic responses or more were noticed, the treatment could be considered effective.

The scientific committee decided to stop the inclusions after the first stage, according to the interim analysis showing zero responders among the first nine patients included. Because of the impossibility of stopping recruitment until the observation of the 3-month primary endpoint, one additional patient was included before the study discontinuation was decided. We present herein the results of the statistical analyses performed on the whole study population (ten patients). Continuous variables were described as standard summary statistics, such as number of observations, median, and range. Categorical variables were summarized in frequency tables as counts and percentages. Time to skeletal-related events was calculated as the interval of time between inclusion and the occurrence of skeletal-related events or the last follow-up date if no skeletal-related event occurred. Overall survival (OS) was defined as time from inclusion to death due to any cause or the date of the last follow-up for alive patients. Time-to-event variables were estimated using the non-parametric Kaplan-Meier method.

## Results

**Fig. 1:**
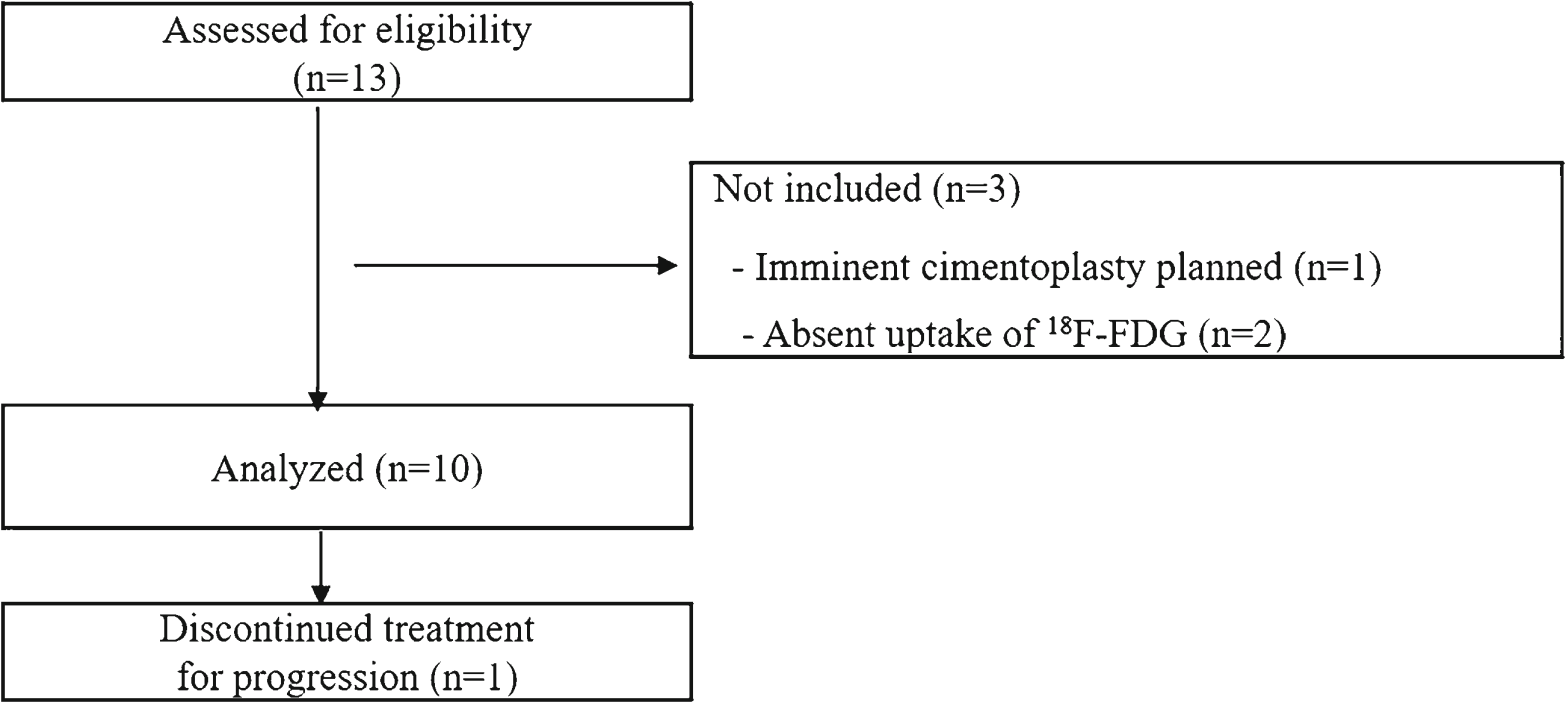
Consolidated standards of reporting trials (CONSORT) DIAGRAM of the study

A total of 13 patients were assessed for eligibility (Fig. 1). Three screened patients failed because of the absence of ^18^F-FDG uptake at baseline (*n* = 2) or because of imminent cimentoplasty needed (*n* = 1).

Ten patients were enrolled between July 2015 and December 2017 (4M/6F; median age 74 years), at Gustave Roussy (*n* = 5), at Institut Universitaire du Cancer Toulouse Oncopole (*n* = 3) and at Groupement Hospitalier Est of Bron (*n* = 2). The main initial characteristics of the patients are presented in Table 1. The median time since the first bone metastasis discovery was 6 years. They received a median of 4 RAI administrations before inclusion, with a median cumulated activity of 15 GBq (range: 7.4–35 GBq). At least one previous bone treatment had been given to each patient consisting of external radiation therapy (*n* = 9), bone surgery (*n* = 8), cimentoplasty (*n* = 5), and cryoablation (*n* = 2). Three patients were treated with bisphosphonates and two with RANK-ligand inhibitors prior to inclusion. None of the patients received tyrosine kinase inhibitors prior to inclusion.

**Table 1:** Patients characteristics at inclusion

Median age at inclusion (years)	74 [46–88]
*Sex*	4 males (40%) / 6 women (60%)
*Histology*	
*Papillary*	5 (50%)
*Follicular*	2 (20%)
*Hurthle cell*	1 (10%)
*Poorly differentiated*	2 (20%)
*ECOG score*	
*0*	5 (50%)
*1*	4 (40%)
*2*	1 (10%)
*Median time since total thyroidectomy (years)*	7 [1–16]
*Median delay since discovery of 1st bone metastases (years)*	6 [0–17]
*Median time since last* ^*131*^*I treatment (years)*	3 [0–10]
*Median number of* ^*131*^*I treatments*	4 [2–8]
*Median total cumulated activity (GBq)*	15 [7.4–35.6]
*Treatment of bone metastases*	
*External radiation*	9 (90%)
*Surgery*	8 (80%)
*Cementoplasty*	5 (30%)
*Cryoablation*	2 (20%)
*Bisphosphonates*	3 (30%)
*Rank ligand inhibitors*	2 (20%)
*Presence of visceral metastases at inclusion*	
*Neck (lymph nodes)*	1 (10%)
*Lung*	5 (50%)
*Localization of bone metastases*	
*Axial*	10 (100%)
*Peripheral skeleton*	3 (30%)
*Number of patients according to target lesion type*	
*Only lytic*	5 (50%)
*Only mixed*	1 (10%
*Lytic + mixed*	2 (20%)
*Lytic + sclerotic + mixed*	2 (20%)
*Patients with negative Ab anti Tg*	7
*Median Tg levels at baseline (ng/ml)*	14.645 [486–23.200]
*Patients with positive Ab anti Tg*	3
*Median value of Abanti Tg (> 4 UI/mL)*	25 [8–25]
*Median ALP levels at baseline (UI/L)*	62 [48–122]
*Median b-ALP levels at baseline (U/L)*	8 [0–22]

The results are reported by median (range)

*ECOG* Eastern Cooperative Oncology Group, *Tg* thyroglobulin, *ALP* alkaline phosphatase, *Ab* antibodies

### Treatment

Nine of the 10 patients received six cycles of Radium-223, whereas Radium-223 treatment was stopped after three cycles in one patient because surgery was then indicated for a skeletal event.

### Metabolic response

**Fig. 2:**
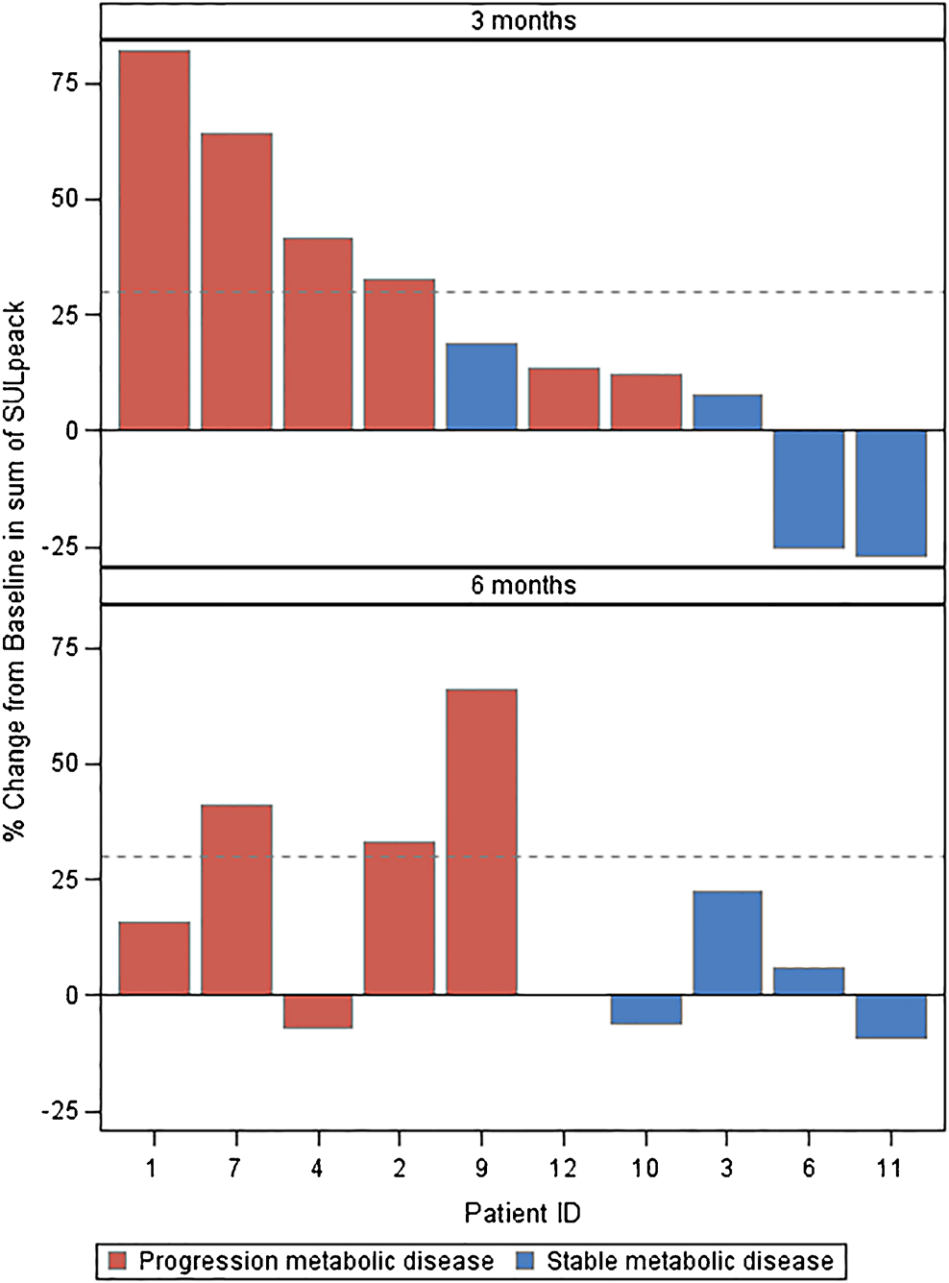
Waterfall plot of total SULpeak variation at 3 months and at 6 months for each included patient according to response criteria used for ^18^F-FDG PET/CT. Disease progression (red column) was defined also in case of SULpeak increase less than 30% but increase > 75% of total lesion glycolysis (TLG) or in case of appearance of new lesions

At baseline ^18^F-FDG PET/CT, a median number of 4 lesions (range 1–11) including axial and peripheral skeleton was detected. Eight out of ten patients had less than 10 lesions (10 and 11, respectively, in the two remaining cases). A total of 28 bone lesions (range: 1–5), in accordance with the target lesions selection criteria, were evaluable on ^18^F-FDG PET/CT for SUL peak and TLG calculation according to PERCIST and considered for analysis. Among the 28 lesions, 15 were classified as lytic lesions, 10 as mixed lesion, and 2 as sclerotic lesions and 1 was not clearly visible on CT but with high ^18^FDG uptake. The median baseline SUL peak per lesion was 3.2 (range: 1.1–29.6) and the median total SULpeak was 10.0 (2.2–51.6); the median TLG for lesion was 21.3 (range :1.7– 283) and the median total TLG was 122.8 (range: 13–530), respectively. After three administrations of Radium-223, disease was defined as stable (SD) in 4 cases and progressive (PD) in 6 cases according to SUL peak and/or TLG. After six administrations of Radium-223, in the 9 evaluable patients, response was SD in 4 cases and PD in 5 cases (Fig. 2). One patient (patient *n* = 10) classified as PD for a significant increase of TLG in targets lesions at 3 months was classified as SD at 6 months. Another patient (patient *n* = 4) with both bone and lung lesions was defined as PD at 3 months for a significant increase of uptake in target lesions but as SD at 6 months at a bone level; finally, he was defined as PD for the appearance of a new lung lesion. The remaining 5/6 patients who presented at inclusion distant metastases in other sites (neck and lung) did not show during the 6-month treatment any progression in visceral metastases at ^18^F-FDG PET/CT. Patients classified at 6 months as PD had papillary (*n* = 2), follicular (*n* = 1), poorly differentiated (*n* = 1), and Hurthle cells (*n* = 1) carcinoma, while patients classified as SD had papillary (*n* = 3) and follicular (*n* = 1) carcinoma, respectively. The median cumulative activity of radioactive iodine previously received was 14.8 GBq (range :11.1–25.9) in patients with PD and 24.9 GBq (range:7.4–35.6) in patients with SD, respectively. The patient that received only 3 Radium-223 treatments (patient *n* = 12) because of the occurrence of skeletal event had poorly differentiated carcinoma and received previously a cumulated activity of radioactive iodine of 11.35 GBq. According to the type of lesions and the per-patient response after the 6 administrations of Radium-223, among patients with PD, 3/5 presented lytic target lesions only, 1/5 mixed lesions only, and 1/5 lytic, mixed, and sclerotic lesions, respectively. Among the 4 patients with SD, 2/4 presented lytic lesions only and 2/4 both lytic and mixed lesions. The patient that received only 3 Radium-223 treatments (patient *n* = 12) had lytic, sclerotic, and mixed lesions, respectively. The per-lesion analysis response at ^18^F-FDG PET according to the lytic/sclerotic/mixed status is presented in Table 2. Histology, cumulative received radioactive iodine activity, number and type of target lesions, SUL peak, and TLG variations in a per-patient analysis are presented in Supplementary Table 3.

**Table 2:** Summary of response after 3 and 6 administrations of Radium-223 in the entire cohort of patients in lesion-by-lesion analysis according to PERCIST criteria on ^18^F FDG PET/CT and according to SUVmax on ^18^FNa PET/CT

Variable	Response	Lytic		Sclerotic		Mixed		Not interpretable	
		^18^FDG PET	^18^FNa PET	^18^FDG PET	^18^FNa PET	^18^FDG PET	^18^FNa PET	^18^FDG PET	^18^FNa PET
*3 months evaluation*	Number	15	17	2	1	10	10	1	1
	PR	1	0	0	0	1	0	0	0
	SD	8	15	1	1	8	10	1	1
	PD	6	2	1	0	1	0	0	0
	Number	12	15	1	1	9	10	1	1
6 months* evaluation	PR	0	2	0	0	0	2	0	0
	SD	9	13	1	1	5	8	0	1
	PD	3	0	0	0	4	0	1	0

*PERCIST* PET Response Criteria In Solid Tumor

*1 patient was not evaluable at 6 months and the total number of lesions is reduced from 28 to 23 lesions between 3 and 6 months at ^18^F-FDG PET/CT and from 29 to 27 at ^18^F-FNa PET/CT

**Fig. 3:**
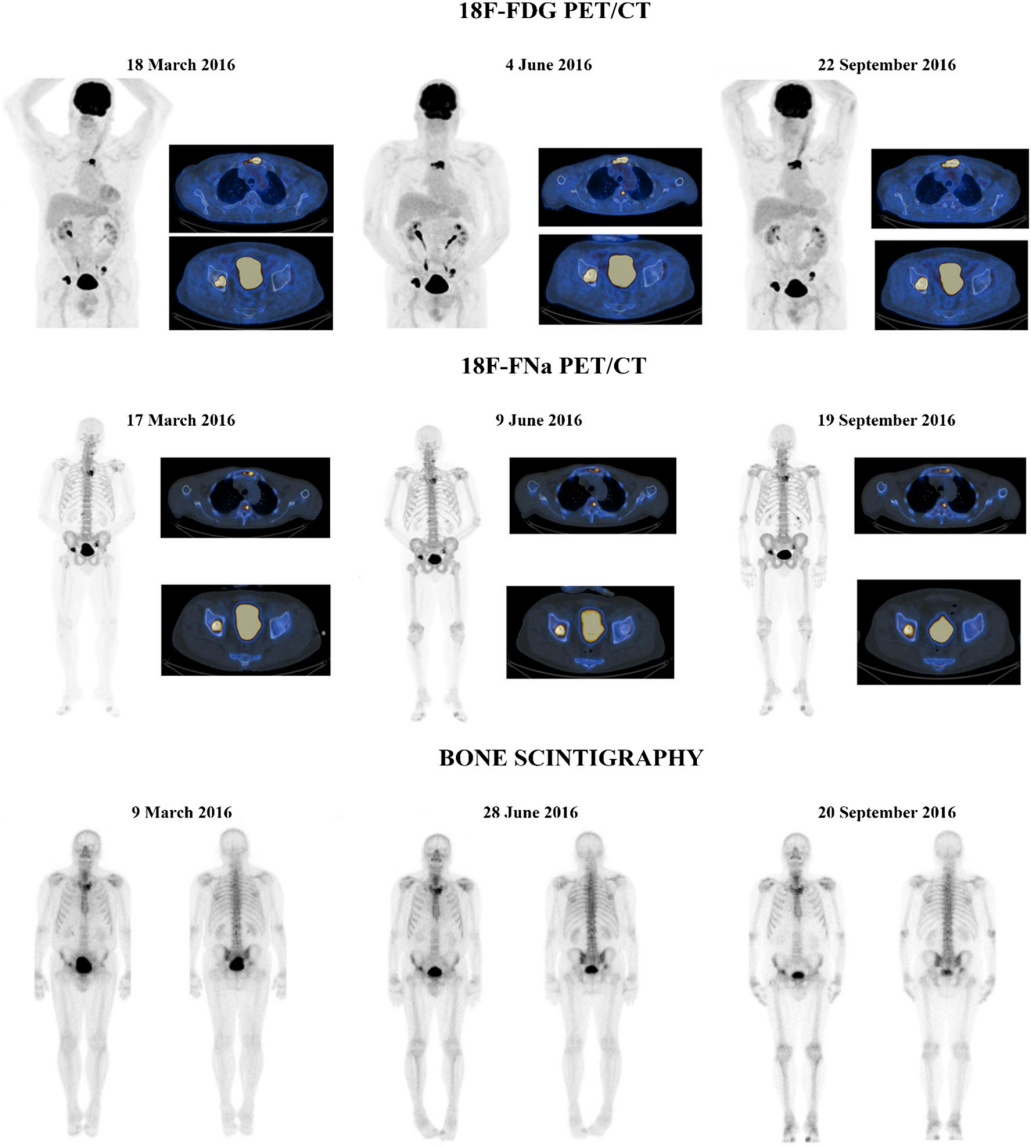
Baseline, 3 months and 6 months. (A) ^18^F-FDG PET/CT. (B) ^18^FNa PET/CT. and (C) ^99^mTc-HMDP bone scintigraphy. The patient presented at baseline 4 bone target lesions. Total SULpeak and total TLG at baseline ^18^F FDG PET were 39.1 and 335.3, respectively. He was defined as PD for ∆SULpeak of + 33% at 3 and 6 months (∆TLG of + 51% and + 72%, respectively). Total SUVmax at baseline ^18^FNa PET/CT was 107.4. He was defined with SD with ∆SUVmax of − 21.5% and − 22% after 3 and 6 administrations of Radium-223, respectively. Disease was considered stable at bone scintigraphy both at 3 and 6 months

Twenty-nine lesions were considered target lesions for SUVmax evaluation at ^18^FNa PET/CT. The baseline median SUVmax for lesion was 17 (range: 4.8–41.6) and the median total SUVmax was 48.9 (range:15.7–123.2). After three administrations of Radium-223, tumor response was SD in nine cases and PD in one. After six administrations of Radium-223 in the nine evaluable patients, tumor response was SD in eight cases and PR in one. The only patient classified as PD at 3 months became SD at 6 months (patient *n* = 4) and one patient classified as SD at 3 months presented a PR at 6 months (patient *n* = 11). Both patients presented with lytic lesion only. A comparison between ^18^F-FDG PET/CT and ^18^FNa PET/CT responses is summarized in Supplementary Table 4. After three administrations, ^18^F-FDG and ^18^FNA PET/CT were concordant in five cases (4 SD and 1 PD) and discordant in the remaining five cases (5 PD at ^18^F-FDG PET classified as SD at ^18^FNa PET/CT) (Fig. 3). After six administrations, ^18^F-FDG and ^18^FNa PET/CT were concordant in three cases (all SD) and discordant in the remaining six cases (5 PD at ^18^F-FDG PET/CT classified as SD at ^18^FNa PET/CT and 1 SD at ^18^F-FDG PET classified as PR at ^18^FNa PET/CT). The per-lesion analysis response at ^18^F-FNa PET according to the lytic/sclerotic/mixed status is presented in Table 2. Regarding bone scan, the same target lesions analyzed at ^18^F-FDG PET were considered and all presented a moderate/intense tracer uptake. Tumor response after three and six treatments of Radium-223 was SD in all the cases.

### Clinical benefits

A complete disappearance of pain was observed in three among the eight evaluable patients after three administrations of Radium-223 and in two among the seven evaluable patients after six administrations, whereas the remaining patients had no clinical response. Analgesic drugs were stopped in two of the seven treated patients after three administrations of Radium-223 and were continued otherwise. ECOG status showed an improvement of at least one point in two patients after three and six Radium-223 administrations, whereas it remained stable in the other patients.

### Biological markers

Among the seven patients without Tg antibodies, a significant decrease (> 50%) in Tg level was observed in three cases after one, four, and six administrations of Radium-223. During follow-up, no prolonged response according to Tg levels was reported. At 3 months follow-up, Tg levels were stable compared to baseline in 5 patients and progressive compared to baseline in 2 patients. ALP and b-ALP available in seven patients were in the normal range at baseline in all cases (Table 1). Their levels decreased significantly in three cases; none of them was under treatment with denosumab or bisphosphonate.

### Skeletal-related events

**Fig. 4:**
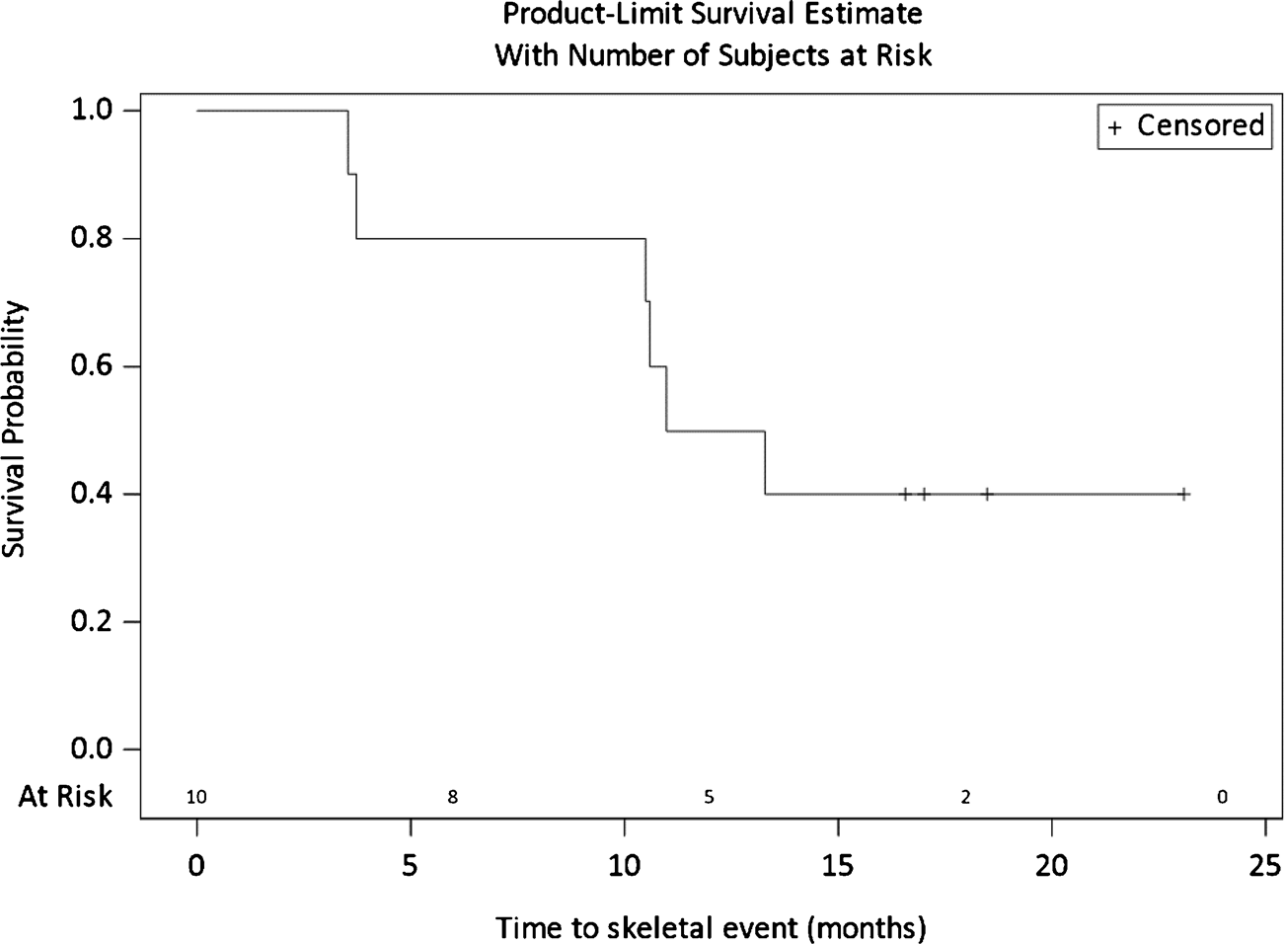
Kaplan-Meier estimation of probability of skeletal event

Skeletal-related events were observed in 6 patients (local progression with indication for local treatment in five cases and pathological fracture in one case), five among them treated with denosumab or bisphosphonate. The median time without skeletal event was 12.1 months (IC 95% 3.5—not evaluable). The Kaplan-Meier curve representing the rate without SRE according to time is presented in Fig. 4.

### Safety

During the 6 months of the Radium-223 treatment period, all patients experienced at least one adverse event. The majority of adverse events (70 of 77 events [91%]) were grade 1–2 events consisting mainly in bone pain (*n* = 8), asthenia (*n* = 8), anemia (*n* = 5), lymphopenia (*n* = 4), leukopenia (*n* = 4), nausea (*n* = 5), pain (*n* = 5), febrile neutropenia (*n* = 3), and thrombocytopenia (*n* = 3). There were seven grade 3 adverse events consisting in lymphopenia (*n* = 2), leukopenia (*n* = 1), febrile neutropenia (*n* = 1), abscess (*n* = 1), thrombocytopenia (*n* = 1), and bone pain (*n* = 1) (Table 3).

**Table 3:** Adverse events occurring during Radium-223 treatment

Grade maximum							
		1		2		3	
Serious	Adverse event	N pat	%	N pat	%	N pat	%
No	Bone pain	8	80	.	.	1	10
	Asthenia	6	60	2	20	.	.
	Lymphopenia	2	20	2	20	2	20
	Leukopenia	3	30	1	10	1	10
	Pain	4	40	1	10	.	.
	Anemia	5	50	.	.	.	.
	Nausea	5	50	.	.	.	.
	Febrile neutropenia	2	20	1	10	1	10
	Thrombocytopenia	3	30	.	.	.	.
	GGT increased	1	10	1	10	.	.
	Sore muscles	2	20	.	.	.	.
	Viral disease	2	20	.	.	.	.
	Diarrhea	2	20	.	.	.	.
	Incontinence	2	20	.	.	.	.
	Abscess	.	.	.	.	1	10
	Bilirubin increased	1	10	.	.	.	.
	Creatinine increased	1	10	.	.	.	.
	HTA	1	10	.	.	.	.
	Hypertriglyceridemia	1	10	.	.	.	.
	Hyperuricemia	1	10	.	.	.	.
	Inguinal hernia surgery	1	10	.	.	.	.
	Neutropenia	1	10	.	.	.	.
	Paresthesia	1	10	.	.	.	.
	Rash pustular	1	10	.	.	.	
	Tachycardia	1	10	.	.	.	
	Urea increased	1	10	.	.	.	
	Vertigo	1	10	.	.	.	
	Alteration of the general condition	1	10	.	.	.	
	Anorexia	1	10	.	.	.	
	Constipation	1	10	.	.	.	
Yes	Thrombocytopenia*	.	.	.		1	10

*Related to acute myeloid leukemia of the patient

In three patients, serious AE occurred after the 6-month treatment period and were reported after the last patient inclusion. These patients developed a myeloid leukemia: one acute myeloid leukemia with a (8;16) cytogenetic translocation, one promyelocitic leukemia with a (15;17) translocation, and one chronic myeloid leukemia with a (9;22) translocation occurred 9, 19, and 8 months after the last Radium-223 administration, respectively (Supplementary Table 5). The first patient (67 years) with an acute myeloid leukemia received prior to enrolment 3 administrations of RAI for a total cumulative activity of 11.1 GBq and one field of external radiation treatment for bone metastases. She was diagnosed 9 months after her last Radium-223 administration and died 4 months after the diagnosis of AML. The second patient (84 years) with a promyelocytic leukemia received prior to enrolment 8 administrations of RAI for a total cumulative activity of 29.6 GBq and 3 fields of external radiation treatments for bone metastases. He was diagnosed 19 months after his last Radium-223 administration and died 2 months after the diagnosis of the leukemia. The third patient (77 years) with a chronic myeloid leukemia received prior to enrolment 5 administrations of RAI for a total cumulative activity of 20.3 GBq and one field of external radiation treatment for bone metastases. He was diagnosed 8 months after his last Radium-223 administration. Three months after diagnosis of chronic myeloid leukemia, he is still alive and being treated with imatinib. All 3 patients presented hematological adverse events during Radium 223 treatment. The first patient presented a grade 3 thrombocytopenia and a grade 2 leukopenia, lymphopenia, and febrile neutropenia. The second patient presented a grade 3 leukopenia, lymphopenia, and febrile neutropenia and a grade 1 thrombocytopenia. The last patient presented a grade 1 leukopenia and neutropenia.

### Overall survival

After a median follow-up of 17.7 months from the last Radium-223 administration, 4/10 patients died. The median overall survival was 21.5 months (IC 95% = [17.5–27.5]), with a 1-year survival of 100% and a 2-year survival of 33%. The reasons for death were promyelocytic leukemia in one case and acute myeloid leukemia in one case. The remaining two patients died because of disease progression 13 and 16 months after the last Radium-223 administration.

## Discussion

In this first trial investigating the efficacy and safety of Radium-223 in patients with bone metastases from RAIR thyroid cancer, there was no evidence of any metabolic response at ^18^FDG PET/CT or significant clinical improvement. According to interim analysis, the trial was stopped after the inclusion of the first ten patients.

Radium-223 has been approved by the FDA and EMA for treatment of patients with symptomatic bone metastases from mCRPC based on ALSYMPCA phase III trial results showing a significant improvement of both overall survival and prolonged time to first symptomatic skeletal events. Recently, the ERA-223 study showed an increased number of skeletal events in patients treated with the association of Radium-223 and abiraterone and it is actually reserved in case of progression after at least two prior lines of systemic therapy for mCRPC (other than LHRH analogues), or ineligible for any available systemic mCRPC treatment [11, 20]. Nevertheless, Radium-223 efficacy in prostate cancer is related to osteoblastic nature of bone metastases, while there are few data on the efficacy of Radium-223 in patients with other cancers with mixed or lytic lesions. Some promising but still low evidence is available in breast cancer patients. In an open-label phase II trial including 23 patients with breast cancer and mainly osteoblastic lesions, Radium-223 significantly reduced uNTX-1 and b-ALP serum levels and induced a 32% metabolic response rate (mRR) on ^18^F-FDG PET [13]. In a second phase II trial including 36 patients with breast cancer treated with Radium-223 in association with hormonal therapy, disease control in 49% of patients and a tumor response rate of 54% assessed by ^18^FDG PET/CT 6 months after last Radium-223 treatment have been reported without grade 3/4 adverse events [16]. In a case report of a single breast cancer patient with diffuse bone metastases, Radium-223 provided bone pain improvement, a decrease in tumor markers with metabolic response at ^18^FDG PET/CT, and at a lower grade at ^18^FNa bone PET/CT [14]. In another case report of a single breast cancer patient with osteolytic metastases, Radium-223 uptake was demonstrated in osteolytic metastases with perfect overlap with the regions of osteolysis previously detected by scintigraphy, indicating a possible therapeutic effect in this setting [15]. Other trials investigating the efficacy of Radium-223 in association with other systemic treatments in non-prostate cancer are ongoing (NCT 02258451, NCT 02258464, NCT 02880943).

Radium-223 is well known to distribute in bone formation area at ^99m^Tc-HMDP bone scintigraphy, and this is the technique of choice to select patients and to monitor response in prostate cancer clinical trials [21]. In our study, ^18^F^-^FDG PET/CT was chosen as principal criteria because most RAI refractory thyroid cancers are ^18^F-FDG avid [22]. ^18^F-FDG PET/CT has indeed been used also for breast cancer trials [13, 16]. Considering SUL peak and TLG as parameters according to PERCIST criteria, no tumor response was observed. PD was seen in 6 cases after three and in 5 cases after six administrations of Radium-223. Otherwise, SD was observed. Even if only SUL peak had been taken into account to define tumor response because of the lower reproducibility of TLG evaluation, there would have been 4 PD and 6 SD after three Radium-223 administrations, which does not change the results of the study.

The distribution of lesions was heterogenous according to disease response in the per-patient evaluation. Among the 28 target lesions considered for analysis at ^18^FDG PET, 15 were lytic, 10 were mixed, and only two were sclerotic and 1 not evaluable. However, the rate of tumor response in mixed and sclerotic lesions was not higher than in lytic lesions, but the small number of lesions analyzed does not allow any firm conclusion. Nevertheless, all the patients were screened before starting treatment to verify the presence of bone remodeling tracers uptake (^18^ FNa PET and ^99m^Tc HMDP) and only patients with ^18^FNa and ^99m^Tc-HMDP uptake in bone lesions were included and considered for Radium-223 treatment.

^18^FNa PET/CT has been proposed to substitute bone scintigraphy to predict and to evaluate the response to treatment in patients with prostate cancer [23]. According to ^18^FNa PET, only one PR was observed after six administrations of Radium-223, whereas on ^99m^Tc HMDP bone scintigraphy, no PR was observed, and the best response was only SD. In summary, ^18^F-FDG PET/CT detected more PD compared to other methods. Furthermore, one case defined as PD at ^18^F-FDG PET after three administrations was finally classified as SD after six administrations, which might be linked to a flare-up phenomenon [24].

Additionally, the clinical benefit was limited with an objective response in pain scale and a decrease in Tg value in three patients. ALP and b-ALP decreased in three cases only, most probably due to a decrease in the physiologic osteoblastic activity since the values of baseline ALP and b-ALP were within the normal range. The rate of skeletal events was also high with six (60%) patients developing a skeletal event, after three administrations of Radium-223 in 1 case and after a median time from inclusion of 12.1 months in the other 5 patients.

According to the safety report, several hematological events have been observed during the treatment period, mainly of grade 1–2. Nevertheless, during follow-up, two acute rapidly fatal myeloid leukemias and one chronic myeloid leukemia were observed. Hematological toxic effects are well known in patients treated with bone-targeting radionuclides [25]. Nevertheless, our data are in contrast with the long-term safety data of patients with prostate cancer treated with Radium-223 [26]. Only sporadic long-term hematological malignancies have been reported, despite dosimetry studies that show that an overall administered activity of 23 MBq in a 70-kg patient after six administrations of Radium-223 would result in an absorbed alpha dose of approximately 1.7–2.0 Gy to the red bone marrow [27, 28]. A major difference with our study is that our patients were previously exposed to high cumulative activities of RAI combined with external beam radiation, but the excess in the relative risk of leukemia reported in such patients is much lower than what is observed in the present study. An excess of relative risk of 0.59 per cumulative activity in GBq of RAI administered was reported in Rubino et al.’s study in patients treated with external radiotherapy [29]. Furthermore, grade 1–3 hematological toxicities during treatment were in favor of a significant effect of bone marrow irradiation by Radium-223. The three leukemias occurred 8 to 19 months after the first Radium-223 treatment, an interval of time in agreement with radiation-induced leukemias observed after the Japanese atomic bombings and after external beam radiation therapy in cancer patients [30]. Based on this interval of time and on the translocations observed, we considered that the leukemias were indeed related to the Radium-223 administrations [31]. The occurrence of secondary blood malignancies that are rapidly fatal is indeed a major concern in the setting of radionuclide therapy [32]. For ^177^Lu-octreotate peptide receptor radionuclide therapy, it ranges in most studies between 1 and 5% but was reported to be as high as 20% in neuroendocrine tumor patients heavily pretreated with alkylating chemotherapy [33, 34].

Even if the nature of the study is prospective, some limitations should be taken into account. First, it included a small number of patients with limited statistical analysis. Regarding the limited number of included patients, the trial was stopped according to the initial design due to the absence of response to Radium-223 at the interim analysis. First, the observation of severe hematological toxicity after the last inclusion in these heavily pretreated patients supported accrual closure. Second, the occurrence of hematological toxicities has been probably underestimated in patients receiving subsequent treatment with alpha emitters after radioactive iodine and external radiotherapy, but it is in our opinion an important information for future studies in the field of radiometabolic treatment. Finally, the criteria used for disease response such as PERCIST in ^18^FDG PET could not be the most appropriate for bone-seeking radiopharmaceutical efficacy evaluation. On the other hand, considering ^18^FNa PET/CT and bone scintigraphy results, only 1 partial response and stable disease in all remaining cases were reported confirming the absence of Radium-223 efficacy in this subset of patients.

## Conclusions

This trial shows the absence of efficacy of Radium-223 in patients with bone lesions from thyroid cancer and a high incidence of hematological leukemia toxicity in heavily pretreated patients.

## Supplementary information

Supplementary Table 1 Inclusion and exclusion criteria in RADTHYR trial. (DOCX 38 kb)

Supplementary Table 2 Response criteria used for ^18^F-FDG PET/CT, ^18^FNa PET/CT, ^99m^Tc HMPD scintigraphy, tumoral markers, bone markers, and pain. (DOCX 31 kb)

Supplementary Table 3 Histology, cumulative received radioactive iodine activity, number and type of target lesions, SUL peak, and TLG variations at ^18^F-FDG PET/CT in a per-patient analysis. In gray color, the box demonstrating progressive disease (PD). (DOCX 12.5 kb)

Supplementary Table 4 Comparison between tumor response on ^18^F-FDG PET and ^18^ FNa PET in a per-patient analysis. (DOCX 24 kb)

Supplementary Table 5 Karyotype of leukemia developed after last Radium223 treatment. (DOCX 27 kb)
