# Supplementary material for: RADTHYR: an open-label, single-arm, prospective multicenter phase II trial of Radium-223 for the treatment of bone metastases from radioactive iodine refractory differentiated thyroid cancer
Source: Eur J Nucl Med Mol Imaging. 2021 Feb 23;48(10):3238–49. doi: 10.1007/s00259-021-05229-y (PMC8426251; doi:10.1007/s00259-021-05229-y)
Supplement: Supplementary file 1 — Inclusion and exclusion criteria in RADTHYR trial. (DOCX 38 kb) [file 259_2021_5229_MOESM1_ESM.docx]

**Supplementary Table 1. Inclusion and exclusion criteria in RAD-THYR trial.**

| **INCLUSION CRITERIA** |  |
| --- | --- |
| Affiliation to a social security regimen |  |
| Histologically confirmed differentiated thyroid cancer (DTC) | Papillary  Follicular including Hurtle cell  Poorly differentiated |
| Radioactive Iodine refractory disease | Definition  - absence of Radioactive iodine (RAI) uptake in metastatic lesions at diagnostic ^131^I scan or after a post-therapeutic ^131^I scan performed several days after RAI treatment or  - in case of RAI uptake present in some but not in other tumor foci or  -progression of the disease within 14 months after RAI administration treatment or  - persistent disease after the administration of a cumulative activity of 22 GBq |
| Age | ≥18 years |
| Eastern Cooperative Oncology Group performance | 0-2 |
| Life expectancy | >3 months |
| Patient fully informed able to comply with the protocol. |  |
| Low likelihood of an indication for systemic treatment within the next 6 months | Defined by:  - absence of soft tissue distant metastases  - presence of only small (<1cm) soft-tissue metastases, or larger (>1 cm) but stable soft tissue metastases within 6 months prior to inclusion |
| Presence of at least one known bone metastasis | Visible on CT scan and not needing imminent local treatment |
| Presence of at least one bone metastasis with increased uptake | ^18^F-FDG PET/CT |
| Presence of at least one bone metastasis with increased uptake | ^99m^Tc HMDP bone scintigraphy or ^18^FNa PET/CT |
| Adequate hematological parameters | -Neutrophils: ≥1,5×109/L  -Platelets: ≥100×109/ L;  -Hemoglobin :> 9g/dL  -Renal function: creatinine <1,5×upper limit of normal range  - Hepatic function: total bilirubin < 1.5 institutional upper limit of normal, aspartate aminotransferase and alanine aminotransferase <2,5×upper limit of normal range in the absence of liver metastases or <5×upper limit of normal range in case of liver metastases |
| Blood negative pregnancy test in women of childbearing potential within 30 days prior to treatment initiation |  |
| Bisphosphonates or anti-RANK ligand (Denosumab) allowed | at least 2 administrations prior to Radium-223 administration and scheduled to be continued during Radium-223 treatment |
| **EXCLUSION CRITERIA** |  |
| Pregnancy or breast feeding |  |
| Another malignancy that was not in remission for at least 2 years before the study | Except for in situ cervix uterine cancer, basocellular skin cancer |
| Concomitant treatment with any investigational drug | To be stopped at least within the previous 4 weeks prior to Radium-223 administration. |
| Imminent or established spinal cord compression immediate need for local radiotherapy | based on clinical findings and/or MRI |
| Progressive visceral metastases | based on RECIST 1.1 criteria assessed by CT scan |
| Symptomatic brain metastases | within 6 months prior to study initiation |
| Symptomatic intestinal disease | Crohn disease or ulcerative colitis |
| Bone marrow dysplasia |  |
| Uncontrolled diabetes or infection |  |
