# Supplementary material for: RADTHYR: an open-label, single-arm, prospective multicenter phase II trial of Radium-223 for the treatment of bone metastases from radioactive iodine refractory differentiated thyroid cancer
Source: Eur J Nucl Med Mol Imaging. 2021 Feb 23;48(10):3238–49. doi: 10.1007/s00259-021-05229-y (PMC8426251; doi:10.1007/s00259-021-05229-y)
Supplement: Supplementary file 2 — Response criteria used for 18F-FDG PET/CT, 18FNa PET/CT, 99mTc HMPD scintigraphy, tumoral markers, bone markers, and pain. (DOCX 31 kb) [file 259_2021_5229_MOESM2_ESM.docx]

**Supplementary Table 2: Response criteria used for ^18^F-FDG PET/CT, ^18^FNa PET/CT, ^99^mTc HMPD scintigraphy, tumoral markers, bone markers and Pain.**

| **METHOD** | **PARAMETER** | **RESPONSE CRITERIA** |
| --- | --- | --- |
| **^18^F-FDG PET/CT** | **SULpeak + TLG** | **Complete metabolic response (CMR):** complete resolution of ^18^F-FDG uptake within measurable target lesions so that it is less than mean liver activity and indistinguishable from surrounding background blood-pool levels.  **Partial metabolic response (PMR):** reduction of minimum 30% in target measurable total tumor SULpeak or absolut drop. Absolute drop in SUL must be at least 0.8 SUL units  **Stable Disease (SD):** not CMR, PMR, or PMD.  **Progression (PMD):** increase of 30 % in total SULpeak of target lesions with 0.8 SUL unit increase from baseline scan or new FDG avid lesions or visible increase in extent of FDG tumor uptake (>75% in TLG volume). |
| **^18^FNa PET/CT** | **SUVmax** | **Complete response (CR):** complete resolution of tracer uptake within measurable target lesions  **Partial response (PR):** reduction of at least 30 % of total SUVmax of target lesions without appearance of any new lesion.  **Stable Disease (SD): not CR, PR and PD**  **Progression (PD):** increase of at least 30 % of total SUVmax of target lesions or appearance of any new lesion. |
| **^99m^Tc HMDP scintigraphy** | **Visual analysis** | **Complete response (CR):** disappearance of tracer uptake in all lesions.  **Partial response (PR):** significant visual reduction of ^99mTc^-HMDP uptake in at least 1 lesion without appearance of new lesion.  **Stable Disease (SD):** absence of tracer uptake modification at visual analysis within target lesions.  **Progression (PD):** appearance of new lesions |
| **Thyroglobulin (ng/ml)** | **Serum** | A reduction ≥ 50 % was considered as significant |
| **Total ALP, bALP** | **Serum** | A reduction ≥ 30 % , confirmed 4 weeks later, was considered as significant |
| **PAIN** | **Numerical Visual Analogue Pain Scale**  **rating from 0-to-10** | **Complete Pain response (CPR):** improvement ≥50%  **Partial Pain Response (PPR):** improvement ≥30% and <50% of the worst pain score compared to baseline. |

PET=Positron Emission Tomography; HMPD= Hydroxymethylene diphosphonate; SUL=standardized uptake lean body mass; TLG= Total Lesion Glycolysis; SUV=standardized uptake value; ALP = Alkaline Phosphatase.
