# Supplementary material for: RADTHYR: an open-label, single-arm, prospective multicenter phase II trial of Radium-223 for the treatment of bone metastases from radioactive iodine refractory differentiated thyroid cancer
Source: Eur J Nucl Med Mol Imaging. 2021 Feb 23;48(10):3238–49. doi: 10.1007/s00259-021-05229-y (PMC8426251; doi:10.1007/s00259-021-05229-y)
Supplement: Supplementary file 3 — Histology, cumulative received radioactive iodine activity, number and type of target lesions, SUL peak, and TLG variations at 18F-FDG PET/CT in a per-patient analysis. In gray color, the box demonstrating progressive disease (PD). (DOCX 12.5 kb) [file 259_2021_5229_MOESM3_ESM.docx]

**Supplementary Table 3. Histology, cumulative received radioactive iodine activity, number and type of target lesions, SUL peak and TLG variations at ^18^F-FDG PET/CT in a per-patient analysis. In grey color the box demonstrating progressive disease (PD).**

| N patient | Histology | Cumulative RAI  (GBq) | N target lesions | Type of lesion | Total SULpeak | | | Total TLG | | |
| --- | --- | --- | --- | --- | --- | --- | --- | --- | --- | --- |
|  |  |  |  |  | T0 | ∆3M (%) | ∆6M (%) | T0 | ∆3M (%) | ∆6M (%) |
| 1 | Poorly differentiated | 14.8 | 1 | Lytic | 9.4 | +82 | +16 | 39.2 | +124 | +83 |
| 2 | Follicular | 25.9 | 4 | Lytic=2  Sclerotic=1 Mixed=1 | 39.1 | +33 | +33 | 335.3 | +51 | +72 |
| 3 | Follicular | 29.6 | 5 | Lytic=3  Mixed=2 | 10.6 | +8 | +23 | 80.6 | -8 | +49 |
| 4 | Papillary | 14.8 | 2 | Lytic=2 | 5.3 | +42* | -7* | 36.7 | +53 | +35 |
| 6 | Papillary | 7.4 | 5 | Lytic=1  Mixed=4 | 28 | -25 | +6 | 204.0 | +8 | +24 |
| 7 | Hurtle cells | 11.1 | 1 | Lytic | 2.2 | +64 | +41 | 22.8 | +72 | +128 |
| 9 | Papillary | 14.8 | 3 | Mixed= 2  Not evaluable=1 | 19.0 | +19 | +66 | 164.9 | +27 | +66 |
| 10 | Papillary | 35.6 | 1 | Lytic=1 | 3.3 | +12 | -6 | 170.1 | +92 | +30 |
| 11 | Papillary | 20.3 | 1 | Lytic=1 | 2.2 | -27 | -9 | 13.0 | +22 | +27 |
| 12 | Poorly differentiated | 11.35 | 5 | Lytic=3  Sclerotic=1  Mixed=1 | 51.6 | +13.4 | NA˚ | 530 | +76 | NA˚ |
|  |  |  | 28 |  |  |  |  |  |  |  |

***PD for new lesion in the lung**

**˚NA=not available**

**Abbreviations:** SUL= Lean body mass corrected Standardized Uptake Value; TLG= Total Lesion Glycolysis; ^18^F FDG =^18^F-Fluorodeoxyglucose; PET/CT=Positron Emission Tomography/ Computed Tomography
