# Supplementary material for: RADTHYR: an open-label, single-arm, prospective multicenter phase II trial of Radium-223 for the treatment of bone metastases from radioactive iodine refractory differentiated thyroid cancer
Source: Eur J Nucl Med Mol Imaging. 2021 Feb 23;48(10):3238–49. doi: 10.1007/s00259-021-05229-y (PMC8426251; doi:10.1007/s00259-021-05229-y)
Supplement: Supplementary file 4 — Comparison between tumor response on 18F-FDG PET and 18 FNa PET in a per-patient analysis. (DOCX 24 kb) [file 259_2021_5229_MOESM4_ESM.docx]

**Supplementary Table 4: Comparison between tumor response on ^18^F-FDG PET and ^18^FNa PET in a per-patient analysis.**

|  | **3 months** | | | | **6 months** | | |
| --- | --- | --- | --- | --- | --- | --- | --- |
|  |  | **^18^FDG** | | | **^18^FDG** | | |
|  |  | **SD** | **PD** | **PR** | **SD** | **PD** | **PR** |
| **^18^FNa** | **SD** | **4** | **5** | **0** | **3** | **5** | **0** |
|  | **PD** | **0** | **1** | **0** | **0** | **0** | **0** |
|  | **PR** | **0** | **0** | **0** | **1** | **0** | **0** |

**Abbreviations:** SD=Stable Disease; PD=Progressive Disease; PR= Partial Response; ^18^F FDG =18F-Fluorodeoxyglucose; ^18^FNa = Sodium-Fluoride; PET=Positron Emission Tomography/Computed Tomography
