# Supplementary material for: RADTHYR: an open-label, single-arm, prospective multicenter phase II trial of Radium-223 for the treatment of bone metastases from radioactive iodine refractory differentiated thyroid cancer
Source: Eur J Nucl Med Mol Imaging. 2021 Feb 23;48(10):3238–49. doi: 10.1007/s00259-021-05229-y (PMC8426251; doi:10.1007/s00259-021-05229-y)
Supplement: Supplementary file 5 — Karyotype of leukemia developed after last Radium223 treatment. (DOCX 27 kb) [file 259_2021_5229_MOESM5_ESM.docx]

**Supplementary Table 5. Caryotype of leukaemia developed after last Radium-223 treatment**

| **Patient n°** | **Type of leukaemia** | **Delay after last Radium-223 treatment (months)** | **Previous treatments** | **Caryotype** |
| --- | --- | --- | --- | --- |
| 7 | acute myeloid | 9 | RAI 11.1 GBq + 1 EBR | Cytogenetic translocation (8;16) probably involving the MYST3 and CREBBP genes |
| 3 | promyelocytic | 19 | RAI 29.06 GBq + 3 EBR | Myelogram FISH Translocation (15;17)  Caryotype: translocation (15;17) variant with del 3q5p resulting in a 5p translocation with another unidentified partner and a deletion of 6q large marker |
| 11 | chronic myeloid | 8 | RAI 20.3GBq + 1 EBR | BCR-ABL p210 transcript of type b2a2 and a b3a2 at 30% in the blood  Caryotype: translocation t (9;22) (Philadelphia chromosome) |

Abbreviations: RAI =Radioactive iodine; EBR=External Beam Radiation
